# An STP-HSI index method for urban built-up area extraction based on multi-source remote sensing data

**DOI:** 10.1098/rsos.220597

**Published:** 2022-11-23

**Authors:** Lijing Bu, Dong Dai, Liying Tu, Zhengpeng Zhang, Mingjun Deng, Xinyu Xie

**Affiliations:** ^1^ College of Automation and Electronic Information, Xiangtan University, Xiangtan 411105, People's Republic of China; ^2^ Shenyang Mxnavi Co., Ltd, Shenyang 110000, People's Republic of China

**Keywords:** extraction of built-up area, night-time light remote sensing image, landsat, human settlement index, random forest, multi-source

## Abstract

A change in an urban built-up area can reflect the process of urbanization and the development of a city. At present, multi-source remote sensing data extraction of built-up areas based on the human settlement index (HSI) has achieved relatively good results but the existence of noise, such as light spillover in the night-time light remote sensing data, seriously affects the accuracy of the HSI. In this paper, a high-precision human settlement index (STP-HSI) method based on spatio-temporal remote sensing and point-of-interest (POI) data is presented to improve the classification accuracy in urban built-up areas extractions. First, to correct light spillover, a new night-time light index the fuzzy c-means spatio-temporal point (FCM-STP) based on fuzzy c-means clustering is proposed, which integrates the spatio-temporal characteristics and uses night light video imaging data from Luojia-1 and POI data. Then, based on the FCM-STP index, the HSI is updated to the STP-HSI index. Finally, a random forest algorithm is used to extract the urban built-up areas, and the random forest feature database is composed of normalized difference vegetation index (NDVI), normalized difference built-up index (NDBI) and STP-HSI index features and texture features. To develop and evaluate the accuracy of the new method for built-up areas extraction with multi-source data, three test sites located in the cities of China (Guangzhou, Xiamen and Nanjing) are used. The experimental results show that our method outperforms the single-source multi-spectral (Landsat 8) data extraction results, the overall accuracy is improved by up to 7.52%, and the kappa coefficient is improved by up to 14%. Compared with the HSI index, the maximum contribution rates of the STP-HSI increased by 25.74%. These experimental results show that the method in this paper is feasible.

## 1. Introduction

Urban built-up areas are often used to study the urbanization process of a region, and they are an important indicator of the level of urban development and expansion [1–6]. The data sources for urban built-up area extraction can be specifically divided into those based on optical remote sensing data [7–9] and those based on night-time light remote sensing data [10–13]. In the earliest studies, Shiping *et al*. analysed the target of an image based on high-resolution remote sensing images using a greyscale co-occurrence matrix and the extracted areas were measured with an accuracy of 93.00% [7]. Jun *et al*. extracted urban built-up areas based on Landsat thematic mapper/enhanced thematic mapper (TM/ETM+) images that combined spectral information and multiple textures, which was better than the traditional greyscale co-occurrence matrix [8]. Bhatti *et al*. proposed a built-up area extraction method (BAEM) for areas building extraction using the newer Landsat-8 Operational Land Imager (OLI) data, and the omission and commission errors were reduced by 75.96% and 33.36%, respectively [9]. Due to the image element blending problem in multi-spectral optical remote sensing images, it is difficult to distinguish between built-up areas and non-built-up areas under limited resolution. Night-time light images most directly reflect economic factors, which is conducive to the extraction of urban built-up areas, so the use of night-time light remote sensing images for urban built-up areas has begun to be widely used by scholars. Duque *et al*. performed urban extent delineation by using defence meteorological satellite system (DMSP)/OLS night-time light data [10]. Shi *et al*. compared the results of NPP-VIIRS with those of DMSP for urban built-up areas extraction and concluded that the National Polar Orbit Partnership visible infrared imaging radiometer (NPP-VIIRS) has a higher extraction accuracy [11]. Liu *et al*. used a night-time light remote sensing image of Luojia-1 to extract the built-up areas, and the study found that it had a high spatial resolution and could extract the rich internal information of the built-up areas [12]. Zhang *et al*. used a vegetation index to adjust the urban night-time lights and found that the method could attenuate light spillovers and improve accuracy [13].

The above single-source urban built-up areas extraction methods still have many shortcomings, and many scholars have started to adopt multi-source methods for urban built-up areas extraction. Pandey *et al*. monitored urbanization areas in India by integrating DMSP/OLS night-time lights and SPOT-VGT data, and a support vector machine (SVM) was used to extract urban built-up areas from the mutual calibration datasets in 1998 and 2008 and from the Systeme Probatoire d’Observation de la Terre/Vegetation (SPOT-VGT) dataset [14]. Zhuo *et al*. proposed an extraction method that incorporates night-time lighting data, normalized vegetation index and demographic data and a model that used different urban and rural areas, but there was a problem of negative computational heterogeneity [15]. Tan *et al*. proposed a method to fuse night-time lighting data, digital elevation models, demographic data, road network density and land cover data, which could avoid overfitting by considering more comprehensive factors and had good tolerance for outliers and noise but there was a poor simulation accuracy problem in areas with low or high population densities [16]. Sharma *et al*. proposed an improved methodology for urban built-up areas extraction by combining MODIS multi-spectral data with VIIRS night-time light data, and a region-specific threshold approach was used to extract the urban built-up areas. It was concluded that the resulting map captured detailed information of the urban built-up areas at a global scale that were missed by the existing maps [17]. Guo *et al*. proposed a method to fuse night-time light data, demographic data and building data, which took into account the building distribution and was easy to calculate, but the model was relatively coarse and low precision [18]. Wu *et al*. proposed a method to fuse night-time light data, demographic data, digital elevation model (DEM), land use data and river road network data, which was richer in the considered factors, but the accuracy varied greatly among the different cities [19]. Yang *et al*. proposed a method to extract night-time light data, normalized difference vegetation index (NDVI), enhanced vegetation index, DEM and demographic data, and used a model in urban and rural areas with different methods, which weakened the effects of light saturation as well as light spillover and the model's applicability [20,21]. The above method used multi-source data for urban built-up areas extraction, which can improve the accuracy of urban extraction, but there are still some shortcomings.

At present, the methods for extracting urban built-up areas can be broadly divided into two types, the threshold methods and the machine learning methods. Sirmacek and Unsalan first extracted the edges and corners of buildings in different orientations using Gabor filters and then used these local feature points to vote for candidate urban areas [22]. Li *et al*. constructed the point of interest (POI) and land surface temperature (LST) adjusted NTL urban index (PLANUI) based on the threshold method, fusing POI and surface temperature, and the results showed that the index helps to attenuate the blooming effect of lights [23]. Li *et al*. constructed an index fusing multi-source remote sensing data based on the dichotomous method, and the experimental results showed that the index has a high check-all rate, F1 score and check-accuracy index [24]. However, threshold methods to extract the built-up areas will be disrupted by human factors and is labour intensive. The machine learning methods can achieve automatic segmentation effects and are suitable for the extraction of built-up areas. Bramhe *et al*. used convolutional neural networks for migration learning based on deep learning methods for urban built-up areas extraction [25]. Pal and Mather proposed using SVM for land cover classification and experimentally proved that SVM has a higher classification accuracy than the machine learning (ML) or artificial neural networks (ANN) classifiers [26]. Subsequently, SVM was widely used for built-up areas extraction. Pelizari *et al*. fused multi-sensor features for built-up areas extraction based on a random forest algorithm, and the results showed that the method has a high classification accuracy [27]. Among the machine learning methods, the random forest algorithm is one that contains many decision trees, has a fast computing speed, high accuracy and less overfitting.

In summary, there are currently problems of light spillover and low spatial resolution in the studies for extracting built-up areas based on multi-spectral and hyperspectral remote sensing data such as Landsat. Due to the interference of buildings, vegetation and water bodies, the extraction accuracy of a classification method using the HSI threshold method alone is low. Therefore, a random forest classification method from the perspective of multi-source remote sensing fusion is presented to extract urban built-up areas, with the following research contributions: (i) To reduce light spillover and boundary omission of built-up areas, a comprehensive fuzzy c-means spatio-temporal point (FCM-STP) index is presented that incorporates night-time spatial and temporal information and POI information; night-time lighting data fused with spatial information and time-series information can reduce the light outliers and noise. A night-time lighting data POI has accurate location information and attribute information, allowing the low-light and no-light urban built-up areas to be extracted. (ii) The traditional human settlement index is affected by the low spatial resolution and light spillover of night-time light data, which will reduce the extraction accuracy of the built-up areas. We propose a high-precision human settlement index (STP-HSI) based on the FCM-STP index. (iii) Based on night-time light remote sensing data, Landsat 8 OLI data, POI data and other auxiliary data, a multi-source remote sensing data extraction method for urban built-up areas is presented to improve the extraction accuracy.

## 2. Material and methods

**Figure 1.**
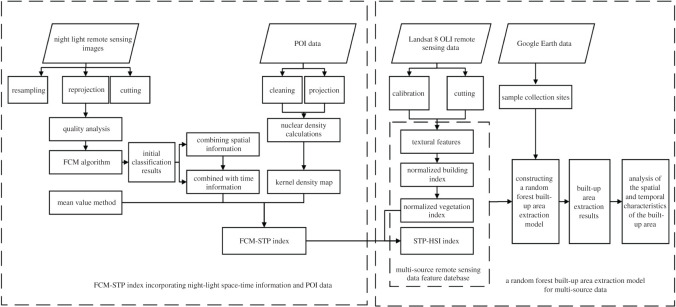
Technology guideline.

The built-up areas extraction method for multi-source data was performed using a random forest algorithm divided into two stages. The FCM-STP index was proposed in the first stage, which is calculated on the basis of the preliminary classification results combining the spatio-temporal information-based night light video imaging data of Luojia-1 and the POI data. In the second stage, the texture features, normalized building index, normalized vegetation index and high-precision human settlement index (STP-HSI) proposed in this paper are composed into a multi-source remote sensing data feature database. The feature database is then used together with the sample points collected from Google Earth data to construct a multi-source random forest built-up areas extraction model for built-up areas extraction. The specific process is shown in figure 1.

### 2.1. FCM-STP night-time light index

Currently, scholars have started to fuse the POI data and night-time light data as a method to extract the urban built-up areas [28], but night-time light data has light spillovers and noise, which leads to fragmented boundaries and scattered patches of built-up areas; and the built-up areas in the low-light and no-light areas cannot be effectively extracted. Starting from the fusion of multi-source heterogeneous spatio-temporal data, to improve the extraction accuracy of urban built-up areas, an FCM-STP comprehensive index that integrates the spatio-temporal features of night-time lights and POIs is proposed, which can effectively use the spatio-temporal features of the night-time lights and POI data.

**Figure 2.**
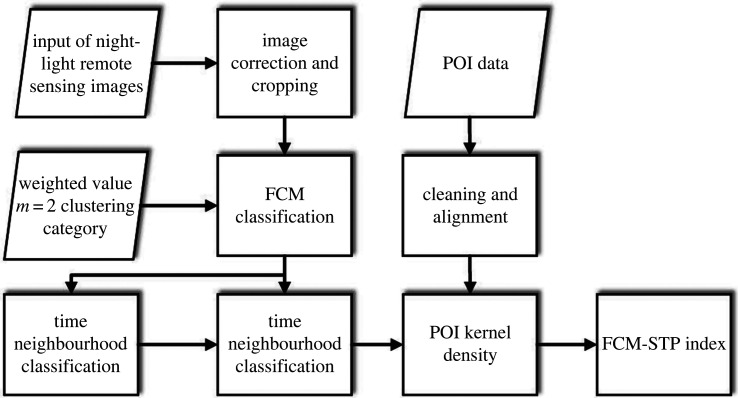
FCM-STP index flow chart.

The index takes into account the fact that FCM is a classical fuzzy clustering algorithm and uses the FCM method for initial automatic classification to avoid some errors caused by unnecessary human factors. Spatial neighbourhood information and time-series information can reduce noise and anomalous data. POI data have accurate location information and attribute information, which can be used to extract the low-light and no-light urban built-up areas. Finally, a comprehensive index of FCM-STP is obtained by fusing information from night-time spatial and temporal data and POI data. The POI data kernel density processing formula, FCM formula [20] and FCM-STP composite index are calculated as shown in formulae (2.1)–(2.3). The flow of FCM-STP index calculation is shown in figure 2.

2.1 $$P_{i}=\frac{1}{n\pi R^{2}}\times \sum_{\;j=1}^{n}K_{j}{\left(1-\frac{D_{ij}^{2}}{R^{2}}\right)}^{2},$$

2.2 $$J=\sum_{i=1}^{c}\sum_{k=1}^{n}{\left(u_{ik}\right)}^{m}{\left\|x_{k}-v_{i}\right\|}^{2}$$

2.3 $$\;\mathrm{and}\;\;\text{FCM-STP}=\sqrt{\frac{\sum_{y=1}^{T}W_{y}(Z_{R}^{i}/Z_{R})}{\sum_{\;j=1}^{c}\sum_{y=1}^{T}W_{y}(Z_{R}^{j}/Z_{R})}\times P_{i}}.$$

In formula (2.1), $K_{j}$ is the weight of data point *j*; $D_{ij}$ is the Euclidean distance between spatial point *i* and data point *j*; *R* is the bandwidth $\left(D_{ij}<R\right)$ of the calculation rule areas; and *n* is the number of *j* data points in the calculation rule areas.

In formula (2.2), $u_{ik}$is the membership degree, the constraint condition satisfies $\sum_{i=1}^{c}u_{ik}=1(1\leq k\leq n)$, $u_{ik}\geq 0(1\leq i\leq c,1\leq k\leq n)$; $\|x_{k}-v_{i}{\|}^{2}$ is the Euclidean distance between the pixel $x_{k}$ and the *i*th cluster centre $v_{i}$; *n* is the total number of pixels in the image to be classified; *c* is the number of cluster categories; and *m* is the fuzzification parameter.

In formula (2.3), $Z_{R}^{i}$ is the number of pixels belonging to the *i*th type in the spatial neighbourhood of the initial urban built-up areas classification result *J* obtained from formula (2.2); $Z_{R}$ is the number of pixels in the spatial neighbourhood; $W_{y}$ is the time weight of the *y*th frame in the *T* frame night-time light data; and $P_{i}$ is the kernel density calculated from the POI data kernel density from formula (2.1).

### 2.2. High precision human settlement index (STP-HSI)

The traditional human settlement index (HSI) helps in urban built-up areas extraction but has certain drawbacks, such as difficulty in distinguishing urban areas from bare soil and water bodies, and low recognition of areas with no or low light [29,30]. Therefore, we made further improvements based on the HSI index principle. Considering that the HSI is calculated from the night-time light remote sensing image and NDVI together [31,32], the original night-time light remote sensing image has problems such as light spillage and noise, which affect the accuracy of the HSI to a certain extent. This paper proposes the STP-HSI index, which improves the scenario when an image element has both built-up and unbuilt-up urban areas by fusing the neighbourhood and time-series information of the image element on the night-time light remote sensing image space with the POI data, thus improving the accuracy of the traditional human settlement index. The STP-HSI takes into account the disadvantages of poor greyscale information and the noise immunity of the individual image elements and the effect of anomalous data on the extraction results. The original HSI formula and NDVI for the normalized vegetation index and the improved high-precision human settlement index are shown in formulae (2.4)–(2.6).

2.4 $$\mathrm{HSI}=\frac{(1-\mathrm{NDVI})+{\mathrm{OLS}}_{\mathrm{nor}}}{(1-{\mathrm{OLS}}_{\mathrm{nor}})+\mathrm{NDVI}+{\mathrm{OLS}}_{\mathrm{nor}}\times \mathrm{NDVI}},$$

2.5 $$\mathrm{NDVI}=\frac{\mathrm{NIR}-\mathrm{Red}}{\mathrm{NIR}+\mathrm{Red}}$$

2.6 $$\begin{array}{r} \mathrm{and}\;\mathrm{STP}-\mathrm{HSI}=\frac{\left(1-\mathrm{NDVI})+((E-E_{\min})/(E_{\max}-E_{\min})\right)}{(1-((E-E_{\min})/(E_{\max}-E_{\min})))+\mathrm{NDVI}+((E-E_{\min})/(E_{\max}-E_{\min}))\times \mathrm{NDVI}}, \end{array}$$

where $\mathrm{OL}S_{\mathrm{nor}}$ is the night-time light index and *E* is the FCM-STP index.

### 2.3. Random forest feature database

In the random forest algorithm [33], the eigenvalues of the feature database have a large impact on the random forest classification accuracy. In this paper, 59 feature values were extracted, taking into account the geographical, architectural and economic factors, specifically including one high-precision human settlement index, STP-HSI, based on multi-source remote sensing data extraction, 58 feature values from the random forest method built-up areas extraction feature database for single-source remote sensing data, 56 texture features, 1 normalized vegetation index and 1 normalized architectural index, as shown in table 1 below.

**Table 1.** Feature parameters of the built-up areas extracted from random forest based on multi-source remote sensing data.

feature classification	name	indicates	number of eigenvalues
texture features	mean	mean_b1,2,3,4,5,6,7	7
	varian	varian_b1,2,3,4,5,6,7	7
	contrast	contrast_b1,2,3,4,5,6,7	7
	homogeneity	homogeneity_b1,2,3,4,5,6,7	7
	dissimilarity	dissimilarity_b1,2,3,4,5,6,7	7
	correlation	correlation_b1,2,3,4,5,6,7	7
	entropy	entropy_b1,2,3,4,5,6,7	7
	second-moment	second-moment_b1,2,3,4,5,6,7	7
feature index	normalized vegetation index	NDVI	1
	normalized building index	NDBI	1
	high precision human settlement index	STP-HSI	1

## 3. Experiment and discussion

To broadly evaluate the superiority of the method proposed in this paper, the method was implemented in three Chinese cities, Guangzhou, Xiamen and Nanjing. The cities were selected to cover different geographical locations and climatic conditions across China. The overall accuracy (calculated from the confusion matrix) and kappa coefficient were used to evaluate the model accuracy. In general, the overall accuracy of the confusion matrix was close to 1, the kappa coefficient is between [0.6, 1], the classification results were highly consistent with the actual results, and the classification accuracy was high. The kappa coefficient was between [0.4, 0.6], the classification result was moderately consistent with the actual result, and the classification accuracy was average.

### 3.1. Datasets and data preprocessing

The datasets used in this experiment were the night-time light remote sensing data, Landsat 8 OLI remote sensing data and POI data, and the datasets were preprocessed accordingly.

#### 3.1.1. Night-time light remote sensing data

Two kinds of night-time light remote sensing data, Luojia-1 and NPP-VIIRS, were selected to evaluate the extraction accuracy of the different night-time light data in the built-up areas.

The ‘Luojia-1’ satellite is widely used in urban built-up areas extraction [34]. Its imaging mode is a frame pushing and sweeping imaging mode, and the sampling interval is 5 s per frame; therefore, there will be multiple night light remote sensing images taken continuously in the same areas on the same day. The main parameters of ‘Luojia-1’ are as shown in table 2.

**Table 2.** Luojia-1 night-time light remote sensing satellite parameters.

parameter items	indicators	parameter items	indicators
time series	June 2018–present	resolution (m)	100–150
nominal height of track (km)	500–600	positioning accuracy (m)	Better than 700
number of image elements	2048 × 2048	SNR (dB)	better than 35
imaging spectrum (nm)	full colour 480–800	revisit time (days)	15

The NPP satellite is a satellite launched by the United States to detect the Earth's environment; it carries a VIIRS sensor with high sensitivity and captures images in both day and night modes. Day/Night Band (DNB) is one of the bands in NPP-VIIRS and is mainly used to detect night light information.

Night-time light remote sensing data preprocessing mainly includes reprojection, resampling and image cropping. The resampling accuracy of the Liaojia-1 night-time light remote sensing image is 50 m, which is consistent with the spatial resolution of the POI kernel density map. Image cropping is performed using Chinese administrative boundary vector data to obtain two types of night-time light remote-sensing images of the desired city. Regarding the preprocessing of the **‘**Luojia-1**’** night-time light remote sensing data as radiation correction, the expression is as follows:

3.1 $${\mathrm{NTL}}_{\mathrm{Luojial}-01}=DN^{3/2}\cdot 10^{-10},$$

where DN and ${\mathrm{NTL}}_{\mathrm{Luojial}-01}$ are the image brightness values before and after a radiation correction.

#### 3.1.2. Landsat 8 Operational Land Imager remote sensing data

Landsat 8 is one of the longest observing Earth satellites, with a large amount of Earth observation data, and it can usually be applied directly without requiring geometric corrections. To use Landsat 8 remote sensing data rich in beam spectral information and to best retain the information in the original spectrum, radiometric calibration and image cropping must be performed [35–37].

#### 3.1.3. Point-of-interest data

**Figure 3.**
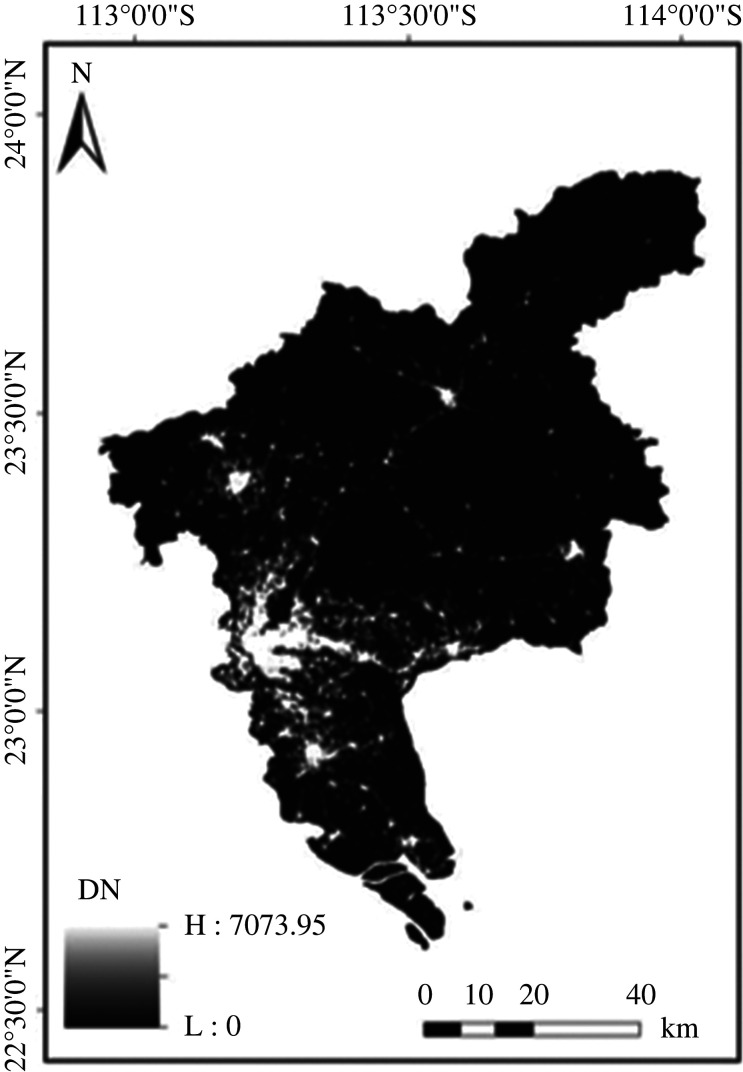
POI kernel density of Guangzhou.

POI data exists in point form, and each point-of-interest datum represents a geographical object. It has high data accuracy, timeliness and rich information, so many scholars also apply POI data to the field of urban planning [38]. In this paper, the POI data of Guangzhou city (2018), Nanjing city (2018) and Xiamen city (2018) were used in our experiments, and the POI kernel density map of Guangzhou is shown in figure 3.

### 3.2. FCM-STP night-time light index results and analysis

**Figure 4.**
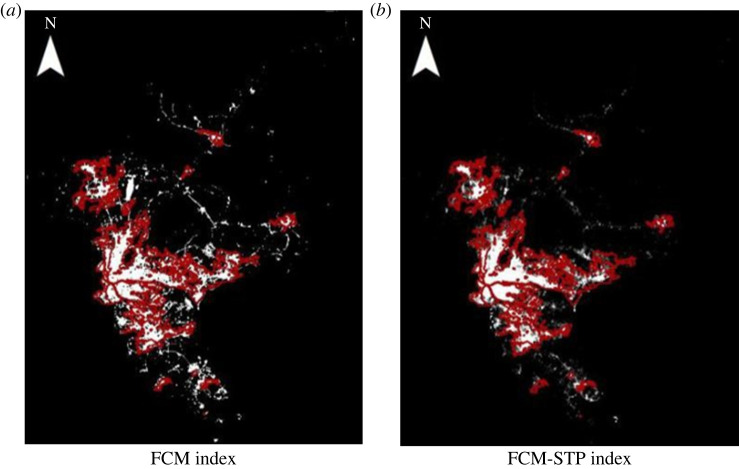
Comparison of the two algorithms based on the referenced built-up areas for Guangzhou city. (*a*) FCM index (*b*) FCM-STP index.

**Figure 5.**
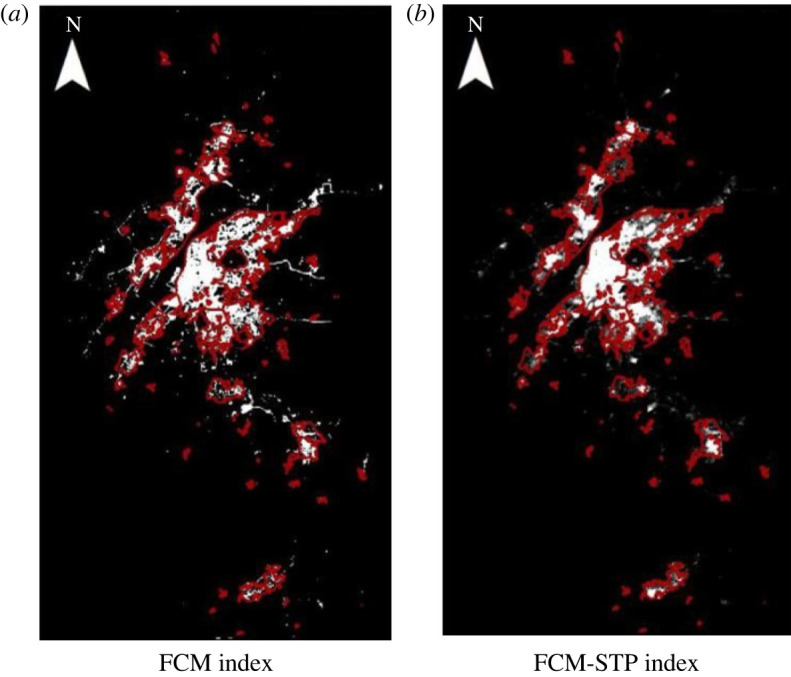
Comparison of the two algorithms based on the referenced built-up areas for Nanjing city. (*a*) FCM index. (*b*) FCM-STP index.

**Figure 6.**
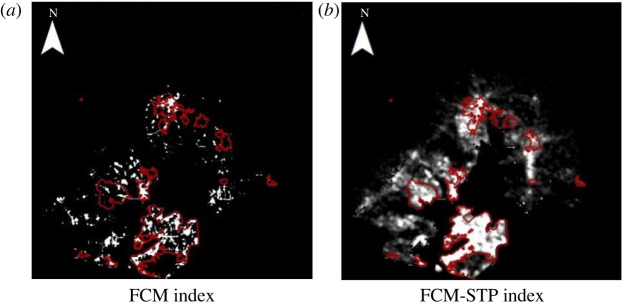
Comparison of the two algorithms based on the referenced built-up areas for Xiamen city. (*a*) FCM Index. (*b*) FCM-STP Index.

To demonstrate the superiority of the FCM-STP index, in this paper, night-time light remote sensing data such as Luojia-1 and NPP-VIIRS were applied. The following experimental results use Luojia-1 as an example. The FCM index method and the FCM-STP index method compared with the reference built-up areas are shown in figures 4–6 (red represents the real data areas, and white represents the index extraction areas). The extraction results show that our proposed FCM-STP index is the closest to the reference built-up areas as a whole, and it can be seen that the lighting data spillover problem is reduced, the influence of lighting data noise on the extraction of built-up areas is reduced and the extracted built-up areas is the closest match to the reference built-up areas. It can be seen that the FCM-STP index proposed in this paper can reduce the light data spillover problem and reduce the influence of light data noise on the built-up areas extraction, which can improve the accuracy of the built-up areas extraction and has universal applicability.

### 3.3. Comparison analysis of built-up areas extracted from single-source and multi-source remote sensing data

**Figure 7.**
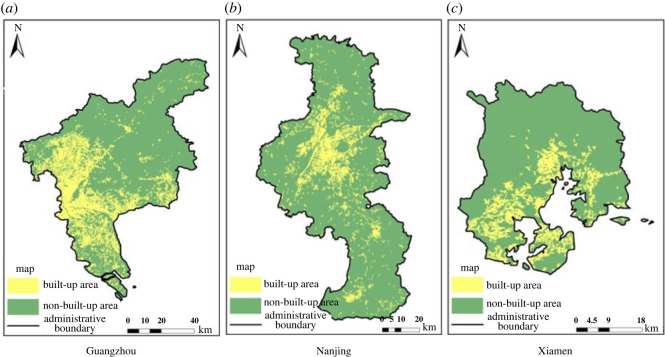
Extraction results of built-up areas based on single-source remote sensing data from Landsat 8. (*a*) Guangzhou. (*b*) Nanjing. (*c*) Xiamen.

**Figure 8.**
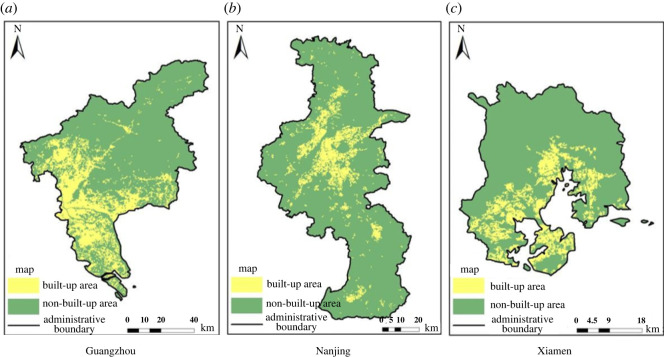
Extraction results of built-up areas based on multi-source remote sensing data from Luojia-1, POI and Landsat 8. (*a*) Guangzhou. (*b*) Nanjing. (*c*) Xiamen.

**Figure 9.**
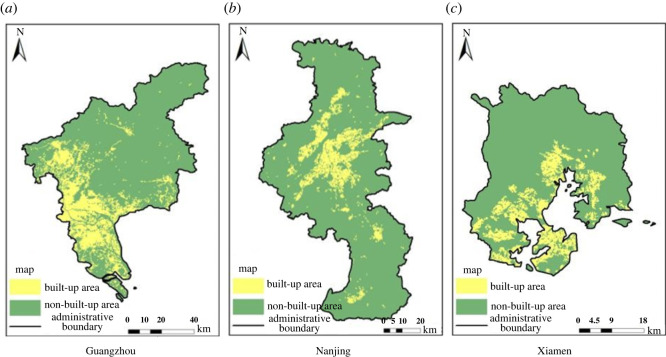
Extraction results of built-up areas based on the multi-source remote sensing data of NPP-VIIRS, POI and Landsat 8. (*a*) Guangzhou. (*b*) Nanjing. (*c*) Xiamen.

In this paper, experiments in three regions are given to compare the accuracy of the built-up areas extraction models based on single-source and multi-source remote sensing data, as shown in figures 7–9, in which the yellow areas indicate the model-extracted urban built-up areas and the green areas indicate the model-extracted non-urban built-up areas. As a result, the built-up areas extraction model simulation map of single-source remote sensing data is generally more patchy, more fragmented and has larger internal gaps than the built-up areas extraction model map of the multi-source remote sensing data, and much information is lost in the extraction process.

The kappa coefficients and the overall accuracy (calculated from the confusion matrix) of the built-up areas obtained for the three test cities are shown in tables 3 and 4.

**Table 3.** Evaluation indices of the built-up areas extraction model based on single remote sensing data.

city	overall accuracy (%)	kappa coefficient
	Landsat 8	
Guangzhou	96.93	0.94
Nanjing	94.62	0.89
Xiamen	94.37	0.89

**Table 4.** Evaluation index of the built-up areas extraction model based on multi-source remote sensing data.

city	overall accuracy (%)		kappa coefficient	
	Luojia-1, POI, Landsat 8	NPP-VIIRS, POI, Landsat 8	Luojia-1, POI, Landsat 8	NPP-VIIRS, POI, Landsat 8
Guangzhou	97.77	98.40	0.96	0.97
Nanjing	95.07	93.72	0.90	0.87
Xiamen	96.54	96.00	0.93	0.92

Comparing the results of the built-up areas extraction model with the single-source and multi-source remote sensing data, it is clearly seen from the accurate evaluation of each group in tables 3 and 4 that the accuracy of the built-up areas model extraction for multi-source remote sensing data is higher than that of the built-up areas model extraction for single-source remote sensing data. The results of the built-up areas extracted by the two methods are used to calculate two evaluation indices, the overall accuracy and the kappa coefficient. The overall accuracy in Guangzhou was improved by up to 1.47%, and the kappa coefficient is improved by up to 3%. The overall accuracy in Nanjing was improved by up to 0.45%, and the kappa coefficient was improved by up to 0.91%. The overall accuracy in Xiamen was improved by up to 2.17%, and the kappa coefficient was improved by up to 4%. In the four groups of model training, the overall accuracy of the results of the built-up areas extraction model training based on multi-source remote sensing data was a minimum of 93.72% and a maximum of 98.40%, the kappa coefficient was a minimum of 0.87 and a maximum of 0.97, the kappa coefficients were all greater than 0.8, and the classification results were almost identical to the actual results. However, the overall accuracy of the training results of the built-up areas extraction model based on single-source remote sensing data was 94.37% at the minimum and 96.93% at the maximum, and the kappa coefficient was 0.89 at the minimum and 0.94 at the maximum. Both indices were lower than the training results of the built-up areas extraction model based on multi-source remote sensing data.

### 3.4. A comparison of random forest-based multi-source extraction methods and support vector machine algorithms

**Figure 10.**
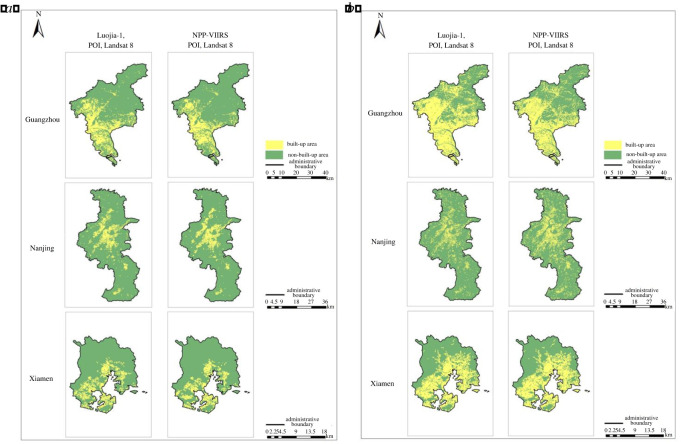
Comparison results of built-up areas extractions based on support vector machine and random forest. (*a*) random forest algorithm. (*b*) SVM algorithm.

To evaluate the superiority of the random forest-based multi-source extraction algorithm and obtain more accurate simulation results of urban construction land, in this paper, three cities, Guangzhou, Xiamen and Nanjing in China, are sampled, and two kinds of night light remote sensing data, Luojia-1 and NPP/VIIRS, are used as data sources for 12 sets of comparison experiments. Simultaneously, the urban built-up areas model based on the random forest method and support vector machine (SVM) simulates 14 sets of experiments in three cities, and after sample validation, the different states presented by different lighting data applied to the same method can be seen in figure 10. Whether applying random forest extraction or support vector machine extraction, it is obvious that the data patches of Luojia-1 star are relatively fragmented, the main urban part of the NPP-VIIRS data patches are distributed in patches with fragmented boundaries, and there is a phenomenon of missing urban boundary information.

Additionally, to evaluate the accuracy of both algorithms, the kappa coefficients and the overall accuracy (calculated from the confusion matrix) of the built-up areas obtained for the three test cities are shown in table 5.

**Table 5.** Evaluation indices of the SVM built-up areas extraction model based on random forest.

city	overall accuracy (%)				Kappa coefficient			
	Luojia-1, POI, Landsat 8		NPP-VIIRS, POI, Landsat 8		Luojia-1, POI, Landsat 8		NPP-VIIRS, POI, Landsat 8	
	random forest	SVM	random forest	SVM	random forest	SVM	random forest	SVM
Guangzhou	97.77	89.48	98.40	93.35	0.96	0.78	0.97	0.86
Nanjing	95.07	84.72	93.72	86.57	0.90	0.69	0.87	0.73
Xiamen	96.54	91.77	96.00	91.77	0.93	0.83	0.92	0.83

Comparing the results of the training of the urban built-up areas extraction model of the random forest algorithm and SVM algorithm, it is clearly seen from the accuracy evaluation of each group that the accuracy of the urban built-up areas extracted by the random forest model is higher than that of the SVM model. The overall accuracy and kappa coefficient were calculated for the results of the built-up areas extracted by the two methods. In 14 groups of model training, the overall accuracy of the SVM algorithm-based urban built-up areas extraction model was 84.72% at the minimum and 93.35% at the maximum, and the kappa coefficient was 0.69 at the minimum and 0.86 at the maximum. The overall accuracy of the results of the random forest algorithm used in this paper is 93.72% at the minimum and 98.40% at the maximum; the kappa coefficient was 0.87 at the minimum and 0.97 at the maximum. Compared with the urban built-up areas extraction model of the SVM algorithm, the results of the random forest algorithm show that the overall accuracy in Guangzhou city was improved by up to 8.29%, and the kappa coefficients were improved by up to 0.18. The overall accuracy in Nanjing was improved by up to 10.35%, and the kappa coefficient was improved by up to 0.21. The overall accuracy in Xiamen was improved by up to 4.77%, and the kappa coefficient was improved by up to 0.10. It can be considered that the method proposed in this paper is better in terms of effect.

### 3.5. Contribution rates analysis of different features

A contribution rate analysis selects some of the feature values, in a dataset with many feature values, that have a large impact on the results by building the model. In this paper, random forest was used to calculate the contribution of the two indices to urban built-up areas extraction. Also, in this paper, the STP-HSI index was presented for urban built-up areas extraction using multi-source remote sensing data. This index has a high contribution rate for the classification of built-up areas under different environmental conditions.

#### 3.5.1. Comparison of the HSI and STP-HSI contributions

**Figure 11.**
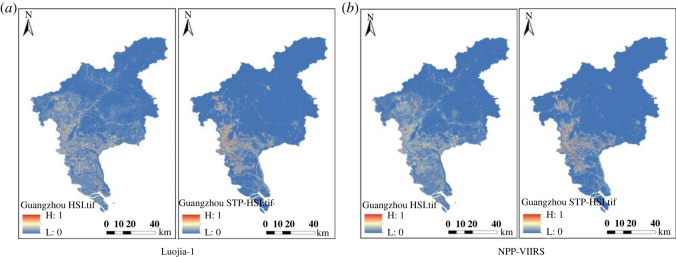
Comparison of HSI and STP-HSI in Guangzhou based on different night-time light data. (*a*) Luojia-1. (*b*) NPP-VIIRS.

**Figure 12.**
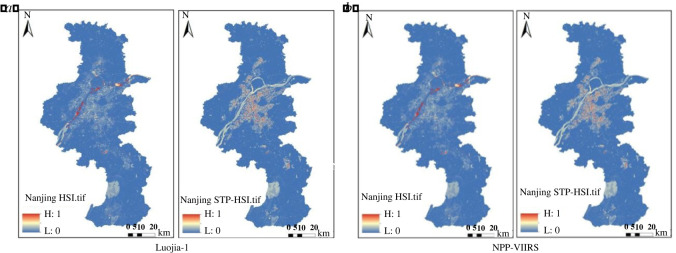
Comparison of HSI and STP-HSI in Nanjing on different night-time light data. (*a*) Luojia-1. (*b*) NPP-VIIRS.

**Figure 13.**
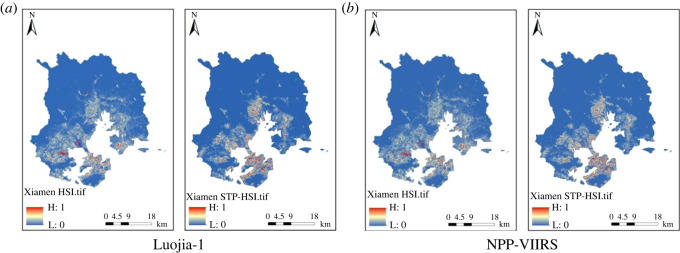
Comparison of HSI and STP-HSI in Xiamen on different night-time light data. (*a*) Luojia-1. (*b*) NPP-VIIRS.

To compare the contribution of HSI and STP-HSI based on the random forest algorithm. In this experiment, STP-HSI and HSI were selected for the construction of the database, and the final contribution rates obtained are shown in table 6. The STP-HSI index of Guangzhou, Nanjing and Xiamen were obtained compared with the traditional HSI index, as shown in figures 11–13.

**Table 6.** Comparison of the contribution rates between HSI and STP-HSI.

city	Luojia-1, POI, Landsat 8		NPP-VIIRS, POI, Landsat 8	
	HSI	STP-HSI	HSI	STP-HSI
Guangzhou	0.4817	0.5184	0.4813	0.5187
Nanjing	0.3713	0.6287	0.4304	0.5696
Xiamen	0.4126	0.5874	0.4618	0.5382

As seen from the comparative plots obtained from the four groups, the range of HSI obtained from the raw night-time light remote sensing data is larger, and the contours of the STP-HSI range are more pronounced. The contribution of the STP-HSI index is generally higher than the contribution of the HSI, as shown by the contribution indicators in table 6. The STP-HSI index contribution was a minimum of 0.5184 and a maximum of 0.6287, while the HSI index contribution was a minimum of 0.3713 and a maximum of 0.4817. In Guangzhou, it improved by up to 3.74%, in Nanjing by up to 25.74% and in Xiamen by up to 17.48%. This reflects the universality of the STP-HSI index and further shows that the FCM-STP index proposed in this paper is useful in increasing the boundary between built and unbuilt areas and reducing light spillover.

#### 3.5.2. Contribution of eigenvalues

**Figure 14.**
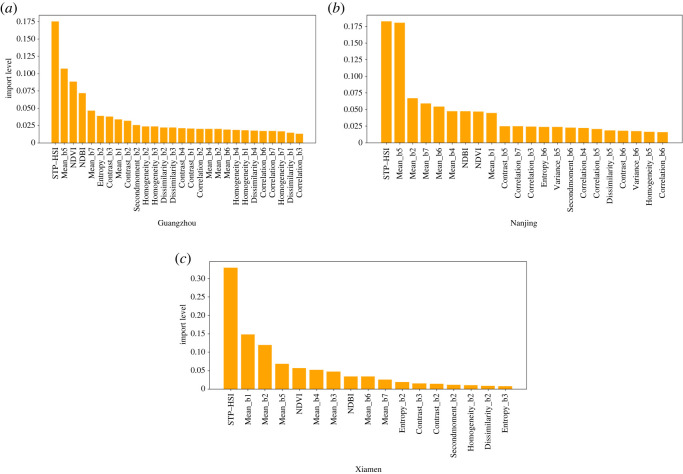
Contribution rates of different features. (*a*) Guangzhou. (*b*) Nanjing. (*c*) Xiamen.

Based on the random forest algorithm, to better extract the urban construction areas and obtain more accurate urban construction areas results in the computing process, this paper sets a feature value greater than 0.01 as the important feature value for this experiment for the built-up areas extraction model feature database. The feature contribution degree graph is shown in figure 14.

From the feature contribution graph, each group of graphs has nine features, including STP-HSI, NDVI, NDBI, Mean_b1, Mean_b2, Mean_b4, Mean_b5, Mean_b6 and Mean_b7, indicating that STP-HSI is normalized. The vegetation index, normalized building index and the mean value of image band texture features have the greatest contribution rates to the extraction of urban built-up areas. The STP-HSI ranks first in the contribution of the 59 eigenvalues, indicating that the human habitation index proposed in this paper is inextricably linked with built-up areas and is an important factor in the extraction of urban built-up areas.

## 4. Conclusion

Remote sensing satellite data offer many new methods for urban built-up areas extraction. In this paper, the main objective was to develop a new method that improves the accuracy of built-up areas extraction. A new STP-HSI index method for urban built-up areas extraction based on multi-source remote sensing data was designed. Considering the shortcomings of the existing methods for urban built-up areas extraction, first, an FCM-STP index is proposed, which can effectively fuse the spatial and temporal information of night-time light and POI attribute information to reduce the light spillover of single-source night-time light data. Furthermore, an STP-HSI was calculated based on the FCM-STP index and added to the random forest database to optimize the extraction results of the urban built-up areas. Experiments have shown that the contribution of the STP-HSI is improved by up to 25.74% compared with the HSI. From the perspective of the extraction method for the random forest method, extracting built-up areas from multiple sources of remote sensing data was better than extracting from single-source remote sensing data, with better overall results and higher model accuracy. Therefore, the application of this method to the extraction of urban built-up areas is of great practical significance.

## Data Availability

Data are available from the Dryad Digital Repository: https://doi.org/10.5061/dryad.qz612jmhr [39].
